# Mortality and nutrition surveys by Non-Governmental organisations. Perspectives from the CE-DAT database

**DOI:** 10.1186/1742-7622-4-11

**Published:** 2007-06-01

**Authors:** Olivier Degomme, Debarati Guha-Sapir

**Affiliations:** 1 WHO Collaborating Centre for Research on the Epidemiology of Disasters (CRED), Université catholique de Louvain, Belgium

## Abstract

In this paper we explore the strengths and gaps among NGO surveys based on an analysis of the records held in the CE-DAT database at CRED. We conclude by recommending the priority areas for strengthening NGO capacity to undertake surveys and ways to improve data quality in general.

## Background

Humanitarian aid has been justified for decades on the basis of its charitable nature rather than its effectiveness. Political expediencies or media coverage has often driven the allocation of emergency aid, recently illustrated by the unprecedented levels of aid for the tsunami disaster compared to that for the Darfur crisis where the humanitarian need was generally considered to be more severe. However, important changes took place during the 1990s.

First, the humanitarian budget grew spectacularly, reaching peaks in the mid 1990s. Today, humanitarian aid budget is close to US$ 10 billion per year and can represent anywhere between 5 – 15% of total ODA in a single donor country. As the budgets increased from early 1990s on, effectiveness and transparency of allocation of these funds came under scrutiny [1].

A second evolution, occurring concurrently with this growth in budget, was the significant increase in the numbers and influence of non-governmental organizations (NGO). From 1600 development NGOs in 1980 registered within OECD industrialized countries, the number had almost doubled in 1993 [2]. By 2000, there were well over 4000 NGOs with nearly half involved in crisis situations.

Due to the absence of national mechanisms to deliver aid and due to a global disenchantment with the public sector in favour of private initiative, NGOs became a major recipient of the increasing budgets. However, the rise in the popularity of NGOs for aid delivery also drew criticisms of poor performance and of NGOs as competitive corporate entities driven more by funding than humanitarian imperatives. The NGOs themselves realized that their capacity to attract support and their legitimacy as actors in humanitarian aid would depend on their ability to demonstrate that they can perform effectively and are accountable for their actions [3]. This pushed them to step up their data collection activities.

In the absence of vital registration systems, typically inexistent or defunct in the regions suffering from the crises, humanitarian workers depend on data obtained through surveys. For many NGOs, this brought about internal reform. The staff profiles changed from a volunteer, often technically unskilled force to an increasingly better paid, better qualified staff. In addition, the need for guidelines and standardized tools to conduct surveys led to initiatives such as the SPHERE project (an NGO initiative) and the SMART program (initiative with NGO membership).

In this paper we explore the strengths and gaps among the surveys conducted by NGOs based on an analysis of the records held in the CE-DAT database at CRED [4]. We conclude by recommending the priority areas for strengthening NGO capacity to undertake surveys and ways to improve data quality in general.

## NGO surveys in CE-DAT: strengths and weaknesses

### CE-DAT: scope, structure and content description

The Complex Emergency Database or CE-DAT is a project within the Centre for Research on the Epidemiology of Disasters (CRED) in Brussels, Belgium. It was created in 2004 in an attempt to collect surveys from complex emergency situations and to make this information available through an internet-based database. The overall objective is to improve evidence-based policy on conflict prevention and response by providing standardized and comprehensive data on the human impact of conflict.

As of January 1^st^, 2007 the CE-DAT database contained information on 1329 nutrition and/or mortality surveys from 41 different countries conducted since 2000 up to November 2006. Surveys are obtained by systematically mining the web, specific sites of humanitarian NGOs, references in literature, PUBMED searches and increasingly through direct agreements for survey report exchanges with UN agencies and NGOs (e.g. Action contre la Faim, Save the Children, Concern, Goal, MSF and IRC). CE-DAT is also the direct recipient of NGO surveys undertaken by partners of USAID-OFDA.

The surveys compiled by CE-DAT are ones that report on the indicators listed in Table 1.

**Table 1:** Indicators for which data is entered in CE-DAT

Mortality indicators	Crude Mortality/Death Rate Under 5 Mortality/Death Rate Infant Mortality Rate Maternal Mortality Rate
Nutrition indicators	Acute Malnutrition (Global, Severe) Chronic Malnutrition (Global, Severe) Underweight (Global, Severe) Oedema MUAC
Vaccination indicators	Measles Polio DTP Tuberculosis

Each survey report is checked for internal consistency, completeness and correct reporting of indicators before entry. The latter are reported in different ways and are standardized for comparability. Details extracted are presented in Table 2. If a few specifications are missing, the survey is included in CE-DAT with a pending status and the producers are contacted for complementary information. In most cases, the response is received quite rapidly. Original reports are archived if available.

**Table 2:** Data from each survey entered in CE-DAT database

Information on the source	Organization(s) in charge of the survey Journal Author Document reference URL
Geographical information	Country Administrative level 1 Administrative level 2 Administrative level 3 City Camp
Information on the methodology	Sample size Sampling Methodology Survey dates Population size % displaced people in the population 95% Confidence interval Mortality recall period
Information on the population	Age group Status (resident, internally displaced, refugee)

Most surveys (over 90%) covered nutritional indicators and almost two thirds of the total covered mortality as well. Vaccination coverage, a key indicator for health protection in crises conditions, was included in half of the cases. (Table 3)

**Table 3:** Percentage of surveys reporting statistics on mortality, nutrition and vaccination coverage

**Indicator covered**	**Number of surveys**	**Percent**
Overall	1329	100,0%
Mortality	845	63.6%
Nutrition	1226	92.2%
Vaccination coverage	716	53.9%

**Figure 1:**
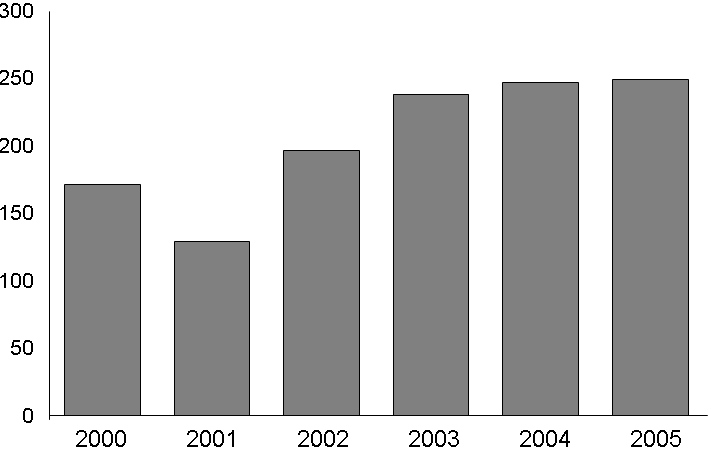
Number of surveys per year entered in CE-DAT.

For 2005, CE-DAT entered 249 surveys, increasing substantially the 169 surveys from 2000 that met its inclusion criteria. This increase may be explained in several ways. First, the number of surveys may have increased over these 5 years. Second, survey reports may be disseminated more widely, making information more accessible. Finally, only surveys reporting specific information are entered in CE-DAT. Since this required information tends to be more often included in more recent reports than it was some years ago, a higher proportion of the surveys now meet the inclusion criteria of CE-DAT. (Figure 1)

### Sources of surveys

Almost all surveys in CE-DAT were undertaken by UN agencies, governments, academic institutions and NGOs either exclusively or in co-operation with each other. We have disaggregated our data according to these source groups. Overall, over two thirds of the surveys were undertaken by NGOs or in partnership with one. UN agencies were involved in 37%. Only a fifth involved government bodies. The distribution is presented in Table 4.

**Table 4:** Percentage and number of surveys conducted by organization category type in CE-DAT

Organization type*	**Percent**	**Number/Total**
NGO	69%	(920/1329)
UN agency	37%	(495/1329)
Government	20%	(264/1329)
Academic	1%	(10/1329)
Red Cross/Crescent	2%	(32/1329)
Other/Unknown	1%	(9/1329)

*Categories not mutually exclusive

**Figure 2:**
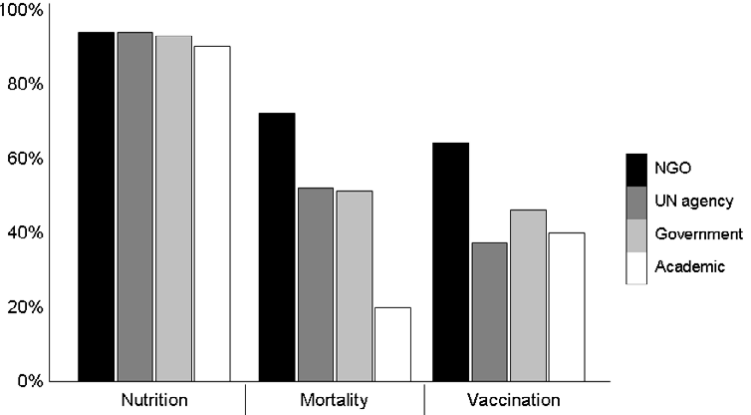
Percentage of the surveys reporting nutrition, mortality or vaccination data by source category.

While almost all surveys, regardless of their source, included nutrition indicators, vaccination and mortality were covered by about half. Only a fifth of the surveys done by academic groups included mortality. It is difficult however to draw any conclusion from this, given the very limited number of academic group surveys (Figure 2).

### Complementarities of geographical coverage

**Figure 3:**
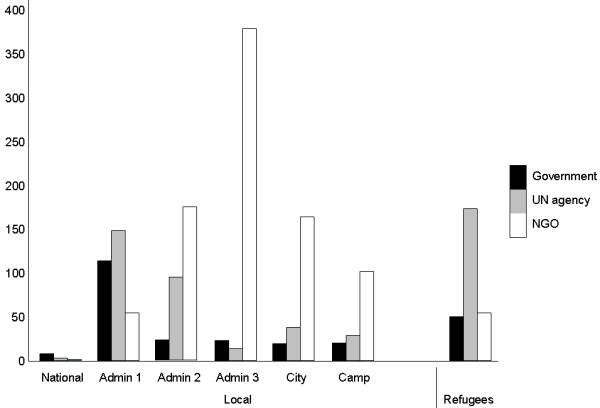
Percent distribution of surveys by organization type and geographical levels.

Surveys undertaken by NGOs cover geographical units at lower resolutions than those undertaken by UN, governments or academic groups. While the UN provides surveys that are nationwide or cover very large regions, NGOs are practically the main source of information at sub national levels for internally displaced people (IDP) and affected residents. In many cases, NGOs have access to insecure areas that for various reasons are inaccessible to academics or UN organizations. (Figure 3)

Data on refugees in camps, on the other hand is primarily available through the UNHCR Standard Indicator Reporting system which partly draws from NGO reports and partly from their own civil registration system [5].

### Quality of NGO surveys

The data selection process in CE-DAT indicates that survey quality and use of standard methodology is more advanced for nutrition sector than for mortality or morbidity. There is consensus on many aspects such as the use of nutritional survey information to confirm the severity of a crisis and on procedures for gathering and analyzing anthropometric data. One should mention however that new growth standards were published by the World Health Organization in 2006 whose use could lead to significant differences in malnutrition figures, especially for severe acute malnutrition [6].

In almost all nutritional surveys the results are triangulated with morbidity and mortality rates, seasonal fluctuations, pre-emergency levels of malnutrition, and the underlying causes of malnutrition. The UNICEF conceptual framework on the causes of malnutrition has been widely adopted as guide in analyzing nutrition problems in populations. Agencies agree on the indicators for monitoring the quality and performance of selective feeding programmes given in the Sphere Project's Humanitarian Charter and Minimum Standards for Disaster Response.

Much of this work on standardization and consensus has been spearheaded by NGOs who frequently sit on various UN Expert Committees.

On the other hand, debate is still open on the optimal methods for measuring mortality and morbidity in crisis situation and their interpretation for judging the severity of the crisis or identifying the appropriate response. Based on the SPHERE guidelines a situation can be labelled "emergency" when the mortality exceeds the double of the peace-time mortality. This would be the case in Iraq. However according to the "1 death per 10,000 people per day" cut-off threshold used by many academics and UN organizations, the situation in Iraq can hardly be called "emergency". Furthermore, surveillance of infectious disease outbreaks, a key element in natural or manmade emergencies, is inadequate leading to either late recognition of outbreaks (e.g. Hepatitis E outbreak in Sudan) or alerts for epidemics that are highly unlikely to occur (e.g. tsunami disaster).

While quality of mortality surveys in conflicts were variable and coverage uneven compared to nutrition, there was little difference between the levels of methodological detail in the survey reports of the NGOs and academic/UN institutions. All sources reported sample sizes, confidence intervals, reference populations and denominators systematically. The original reports of the NGO surveys indicate a careful use of standard methodologies and good understanding of the biases. The reports were usually set out as a research paper with purpose, methods, results, discussion and conclusion.

## Conclusion

Although not all surveys conducted in complex emergencies are included in CE-DAT, we feel that some patterns can be identified when analyzing the more than 1000 surveys included in the database. One has to keep in mind though that this analysis can not necessarily be extrapolated to all existing surveys.

One of the main findings is that NGOs are the main source of surveys from complex emergency sites and are likely to remain so. Besides contributing the bulk of the data, there are important complementarities that the NGOs bring to the larger, more prestigious surveys undertaken by university groups or UN agencies.

First NGOs provide survey information at lower levels of resolution often camps, cities or districts while the UN surveys tend to cover large areas with the inherent problems of generalization in highly heterogeneous situations. Second, NGO surveys aim at assessing a local situation for needs and programming resources (e.g. beneficiaries, food vaccination doses) or for monitoring their impact. UN surveys tend to be large scale snap shots of a situation that serves as a point of reference rather than an operational tool (e.g. UNICEF's Multiple Indicator Cluster Surveys). Third, the NGO surveys provide essential contextual information, such as latest food arrivals or nearby fighting or raids which inform the analyses in volatile conditions. Most academic groups are usually stationed in the area temporarily and are located in less insecure areas. The UN monitors hostilities but as an inter-governmental authority has limitations with regard to presence in insecure parts and access to local information.

Finally, these surveys, whether undertaken by academics, UN or NGOs serve, in the first instance, practical and operational purposes related to priority setting for aid and programme input allocation. In this context therefore, it is important to distinguish quality needed for academic purposes from quality needed for operational purposes.

Although the highest level of scientific quality should be the objective of every study, the main purpose of conducting mortality and nutritional surveys is to provide reliable data rapidly for decision making for humanitarian aid. In this context, one could define a good survey as a survey that leads to a methodologically valid approximation rather than highly precise numbers.

Despite these strengths, the NGO surveys suffer from weaknesses. First consensus on appropriate thresholds and baselines to estimate excess deaths needs resolution, not only for the NGOs but for all the involved parties. Survey findings presented in a reference vacuum is essentially meaningless. Neither can the notion of severity ranking of the crises be useful without the concept of normality. NGOs are frequently the ones that ring alarm bells and alert the wider public to deteriorating situations. Guidelines to place their findings on a recognized scale would make their survey results useable for trend analyses, comparisons with other surveys and for evaluating severity.

Second, NGO survey reports should strengthen the peer review process without compromising on the rapidity of field-to-user turnaround time. The surveys undertaken by UN and research groups are built on sound scientific foundations but at a high cost in timeliness. Research groups typically do not make the survey results public until it has been accepted for scientific publication, easily a delay of 6 to 12 months. On the other hand, limited peer review, as is the case for most NGO surveys, affects both quality and credibility of their work – even if they are comparable to those done by the UN and academics. This being said, NGOs make their results available rapidly and within a useable timeframe. The optimal solution will be to recognize that the shelf life of survey findings in emergency situations is extremely short and a significantly rapid peer review mechanism will have to be found, so the results are not outdated for any practical decision making.

Finally, the paradigm of humanitarian response is shifting fast towards an evidence based response. Accountability and effectiveness of aid are strong motivating forces for donors towards their tax payers and NGOs towards their beneficiaries. NGOs have substantially strengthened their competences and technical skills in gathering credible data and providing evidence on the prevailing humanitarian situation. They are likely to remain the principle source of survey data. It is therefore, in the interests of the humanitarian community and the beneficiaries to further strengthen their capacities.
